# Author Correction: System characterization of a human-sized 3D real-time magnetic particle imaging scanner for cerebral applications

**DOI:** 10.1038/s44172-024-00208-1

**Published:** 2024-04-11

**Authors:** Florian Thieben, Fynn Foerger, Fabian Mohn, Niklas Hackelberg, Marija Boberg, Jan-Philipp Scheel, Martin Möddel, Matthias Graeser, Tobias Knopp

**Affiliations:** 1 Section for Biomedical Imaging, University Medical Center Hamburg-Eppendorf, Hamburg, Germany; 2 Institute for Biomedical Imaging, Hamburg University of Technology, Hamburg, Germany; 3 Fraunhofer IMTE, Fraunhofer Research Institution for Individualized and Cell-based Medical Engineering, Lübeck, Germany; 4 Institute of Medical Engineering, University of Lübeck, Lübeck, Germany

**Keywords:** Biomedical engineering, Imaging techniques

Correction to: *Communications Engineering* 10.1038/s44172-024-00192-6, published online 14 March 2024

Author correction:

In the original version of this Article, financial support from the Open Access Publication Fund of UKE - Universitätsklinikum Hamburg-Eppendorf was not acknowledged. This has now been corrected in both the PDF and HTML versions of the Article.

The Acknowledgements section needs to be revised to:

We thank Florian Sevecke for technical support during the realization of numerous scanner components. We further thankfully acknowledge the financial support by the German Research Foundation (DFG, grant numbers KN 1108/7-1 and GR 5287/2-1). We also thank the developers of Makie.jl supporting us by answering technical questions when creating Figs. 3 and 4. Finally, we are grateful to Christian Findeklee for their discussions and insights into resonant circuit decoupling. We acknowledge financial support from the Open Access Publication Fund of UKE - Universitätsklinikum Hamburg-Eppendorf.

